# Motivational Interviewing As an Adjunct to Periodontal Therapy—A Systematic Review

**DOI:** 10.3389/fpsyg.2017.00279

**Published:** 2017-02-28

**Authors:** Svenja L. Kopp, Christoph A. Ramseier, Petra Ratka-Krüger, Johan P. Woelber

**Affiliations:** ^1^Department of Operative Dentistry and Periodontology. University Freiburg Medical CenterFreiburg im Breisgau, Germany; ^2^Department of Periodontology and Fixed Prosthodontics, University of Bern, School of Dental MedicineBern, Switzerland

**Keywords:** motivational interviewing, motivational intervention, periodontal therapy, periodontal diseases, systematic review

## Abstract

**Aim:** Periodontal therapy is highly dependent on a patient's long-term adherence with regard to oral hygiene, diet, and regular check-ups at the dentist. Motivational Interviewing (MI) is a client-centered, directive method for encouraging a patients' behavioral health change. The aim of this systematic review was to reveal the effects of MI as an adjunct to periodontal therapy.

**Methods:** Three databases (PubMed, Cochrane Library, and Web of Science) were reviewed for randomized controlled clinical trials. Articles were included when using MI as an adjunct to periodontal therapy and presenting clinical periodontal and oral hygiene related parameters. Two authors independently coded the relevant articles.

**Results:** The search yielded 496 articles. After analysis and exclusion, a total of five papers could be included. The quality of the articles ranged between 72–88%. The two independent raters showed a high inter-rater reliability (Cohens-Kappa = 0.89). In two studies MI showed a significant positive effect on bleeding on probing and plaque values. One study showed improvement of self-efficacy in interdental cleaning. Two studies showed no influence of MI on periodontal parameters of the patients.

**Conclusion:** The use of MI as an adjunct to periodontal therapy might have a positive influence on clinical periodontal parameters (plaque values, gingival, and periodontal inflammation) and psychological factors related to oral hygiene (self-efficacy). Due to the low body of evidence further studies are needed. Future studies should include fidelity measures of the applied MI, a high number of counselors, several MI sessions, and long-term study follow-up to show potential effects.

## Introduction

Periodontal disease is one of the major chronic inflammatory diseases of mankind and is affecting about 743 million people worldwide (Kassebaum et al., 2014). Long term success of periodontal therapy crucially depends on a patient's adherence behavior to professional recommendations (e.g., regarding regularly follow-up, smoking cessation, or oral hygiene) (Axelsson et al., 2004; Ramseier, 2005; Eickholz et al., 2008). A current consensus report on principles in preventing periodontal disease emphasized the value of proper oral hygiene and smoking cessation in periodontal practice (Tonetti et al., 2015). Thus, health behavior communication is a key element to support patient's health behavior change in both periodontal therapy and the prevention of periodontitis. Motivational Interviewing (MI, Miller and Rollnick, 2012) is an evidence-based communication method for supporting health behavior change in several fields like weight reduction, smoking cessation, reduction of alcohol consumption, and control of blood sugar (Lundahl et al., 2013; Ekong and Kavookjian, 2016; Jassal et al., 2016). MI is summarily defined as a “collaborative counseling style for strengthening a person's own motivation and commitment to change” (Miller and Rollnick, 2012, 2014). In the field of oral health, MI showed promising effects on preventing caries cavities in children with a high risk of caries and on decreasing dental plaque by improving oral health and oral health knowledge (Godard et al., 2011; Naidu et al., 2015; Albino and Tiwari, 2016; Gauba et al., 2016).

Correspondingly, MI could principally be useful as a tool for motivating patients with periodontal disease. Interestingly, this suggestion was already mentioned in the consensus report on the principles in preventing periodontal disease and the descriptions for the European core curriculum for both undergraduate dental students and postgraduate dental students (Sanz and Meyle, 2010; Tonetti et al., 2015).

A recent systematic review about the use of MI in dental settings by Gao et al. (2014) showed positive, but varying effects of MI especially on improving periodontal health through oral hygiene measurements. Looking closer on the included studies a broad variance could be found regarding the measured indices (plaque, gingival bleeding, psychological parameters, pocket probing depths) and the patients included (from healthy to severe periodontal conditions). Because the review by Gao et al. (2014) aimed to present a wider perspective on MI in dental settings and not as an adjunct to periodontal therapy, a more focused discussion and bias rating regarding the periodontal studies was not performed. Thus, it is still unclear if MI is an evidence-based method for health behavior change in periodontal therapy.

Due to this background, the current systematic review aimed to analyze studies specifically regarding the effect of MI as an adjunct to periodontal therapy.

## Materials and methods

The systematic review was based upon PRISMA-P (preferred reporting items for systematic reviews and meta-analyses protocols) and contained the PICO elements (participants, intervention, comparison, outcomes) for conducting this study (Moher et al., 2015). Three databases, Cochrane Library, Web of Science (Thomson Reuters), and Medline (PubMed), were searched for relevant reports by two independent raters (SLK, CAR). In case of non-available publications Google scholar was checked for access.

The protocol for this systematic review was registered on PROSPERO (Registration Number: CRD42017056450).

The following research questions were targeted for this study:

- Which effect does MI have as an adjunct to periodontal therapy?
- Which effect does MI have on the oral hygiene of the periodontal compromised patient?
- Which effect does the duration of motivational interviewing application have on study outcomes?

### Search strategy

Regarding PICO, the population was defined through the following key words: “periodontitis,” “gingivitis,” “periodontal,” “oral,” “dental,” und “plaque” were connected with the Boolean operator “OR.”

To determine the intervention, the key words “motivational interviewing,” “motivational interview,” and “motivational intervention” were used. These words were connected with the Boolean operator “OR.”

Both, the population and the intervention were connected with the Boolean operator “AND.”

Comparators and outcomes were not included in the search term due to the low number of available studies and in order to receive the maximum of publications.

### Syntax for both databases

(motivational interviewing OR motivational interview OR motivational intervention) AND (periodontitis OR gingivitis OR periodontal OR oral OR dental OR plaque)

Reference lists of included studies were checked for further studies.

Search for ongoing trials or trials completed but not published were conducted in ClinicalTrials.gov and the WHO International Clinical Trials Registry Platform (ICTRP).

The search was performed for studies published before July 2016.

### Data collection

Two authors coded with an inter-rater reliability of 99.80% (Po = 0.9980, Cohens-Kappa = 0.888).

### Inclusion criteria

- Randomized controlled clinical trial
- Patients with periodontal disease
- At least one plaque index (PI) and one inflammatory index (e.g., gingival index—GI, bleeding on probing index—BOP).

### Exclusion criteria

- The use of MI in another area of dentistry than periodontology (e.g., pedodontics)
- The use of MI in other medical areas (alcohol consumption, smoking, drug usage)
- Other study designs than RCTs (e.g., case control studies, cohort studies).

Excluded papers were listed for each database, including the reasons for the exclusion (Table 1).

**Table 1:** **Excluded studies after full text analysis**.

**First author (year of publication)**	**Data source**	**Reason for exclusion**
Lhakhang et al., 2016	E	N2
Harrison, 2014	E	N3
Shamani and Jansson, 2012	E	N1
Godard et al., 2011	E	N2
Yeung, 2010	E	N3
Neves et al., 2015	E	N2
Suresh et al., 2012	E	N2
Halvari and Halvari, 2006	E	N2
Lhakhang et al., 2015	E	N2
Almomani et al., 2009	E	N2
Jönsson et al., 2009a	E	N4
Halvari et al., 2012	E	N2

*E, electronical search of database; N1, not related to MI; N2, not related to periodontal therapy by using GI/ BOP and PI, N3, Commentary, Case report, N4, not controlled*.

### Risk of bias

In this systematic review the risk of bias was examined including the external (e.g., generalization) and internal validity (e.g., reliability). The risk of bias items were based upon Lundahl et al. (2013) regarding the MI-related bias, Schmidt et al. (2014) regarding the periodontal study-related bias, and the German version of the Cochrane Collaboration manual (Schmucker et al., 2016) regarding the general bias. Each item was rated and listed. The ratings of the individual domains are shown for each study in Table 2.

**Table 2:** **Risk of bias assessment**.

**Risk of Bias**		**Woelber et al. (2016)**	**Brand et al. (2013)**	**Stenman et al. (2012)**	**Jönsson et al. (2010)**	**Jönsson et al. (2009b)**
MI-Bias	Type of MI	n.a.	1	1	0	0
	Numbers of counselors	1	0	0	0	0
	MI training of counselors	1	n.a.	n.a.	1	1
	Fidelity measure	1	1	1	1	1
	Dose of MI	1	1	1	0	0
	Number of interventions	1	1	1	1	1
	Profession of counselor	1	0	1	1	1
	Quality of MI	1	n.a.	0	n.a.	n.a.
Perio-Bias	Recruitment of patients	1	1	1	1	1
	Type of periodontal therapy	1	n.a.	1	1	1
	Outcome measures	1	1	1	1	1
	Calibration	1	1	n.a.	0	0
	Check of medication	1	n.a.	n.a.	1	1
	Report of oral hygiene	1	1	1	1	1
	Report of periodontal risk factors (e.g., smoking)	1	1	1	1	1
General bias	Study follow-up	0	0	1	1	1
	Randomisation	0	1	1	1	1
	Blinding	1	1	1	1	1
	Inclusion-/ Exclusion criteria	1	1	0	0	0
	Participants (Exp./ control group)	1	1	1	1	1
	Definition of experimental and control group	1	1	1	1	1
	Number and profession of clinicians	1	1	1	1	1
	Drop Out	1	1	1	1	1
	Funding	1	1	1	1	1
	Results	1	1	1	1	1
Proportion		88%	72%	76%	76%	76%

*Risk of bias assessment adapted from Lundahl et al. (2013), Schmidt et al. (2014) and Cochrane Handbook from Schmucker et al. (2016). 1, Qualitycriteria fulfilled, 0, not fulfilled, n.a., not applicable*.

The MI-related bias assessment intended to provide a qualitative overview of the application of MI (Miller and Rollnick, 1991, 2002, 2012; Lundahl et al., 2013). Accordingly, the following quality factors were checked:

- The MI version should be mentioned (Miller and Rollnick, 1991, 2002, 2012)
- To avoid personal influences (like sympathy, friendliness), it is important to perform the interventions by a high number of counselors
- The duration and number of interventions should be given to indicate the intensity of MI
- The training of the counselors should be mentioned
- The quality of the interventions should be assessed to evaluate the quality and application of MI.

The periodontal study-related bias provides the bias related to periodontal therapy. Following factors were checked (Schmidt et al., 2014):

- Report of the patients' selection whether the patients were diagnosed with chronic periodontitis
- Report of the periodontal therapy (non-surgical periodontal therapy or surgical periodontal therapy)
- Report about the evaluated indices
- Report or control of periodontal risk factors (e.g., smoking status, intake of medication, diabetes)
- Calibration of the rater regarding the clinical measurements.

The general bias report was about the reliability and validity of the study results. This included the following factors (Schmucker et al., 2016):

- Study design, randomization, and blinding
- Comparability of the patient groups to avoid different group characteristics
- Definition of the experimental and control groups
- The number of therapists report of the equal clinical measurements without distortion due to different measurement techniques. Furthermore, the studies should present all significant results and the funding in a transparent manner.

Each item of the rating scale was rated with one point when meeting the criteria. If the information was not given in the study, it was rated with “not applicable” (n.a.). When not meeting the criteria the item was rated with zero points. At the end, a percentaged total score was built (Schmucker et al., 2016). The risk of bias checklist with its criteria is available under the supplementary file “Data Sheet 2.”

## Results

### Screening

**Figure 1:**
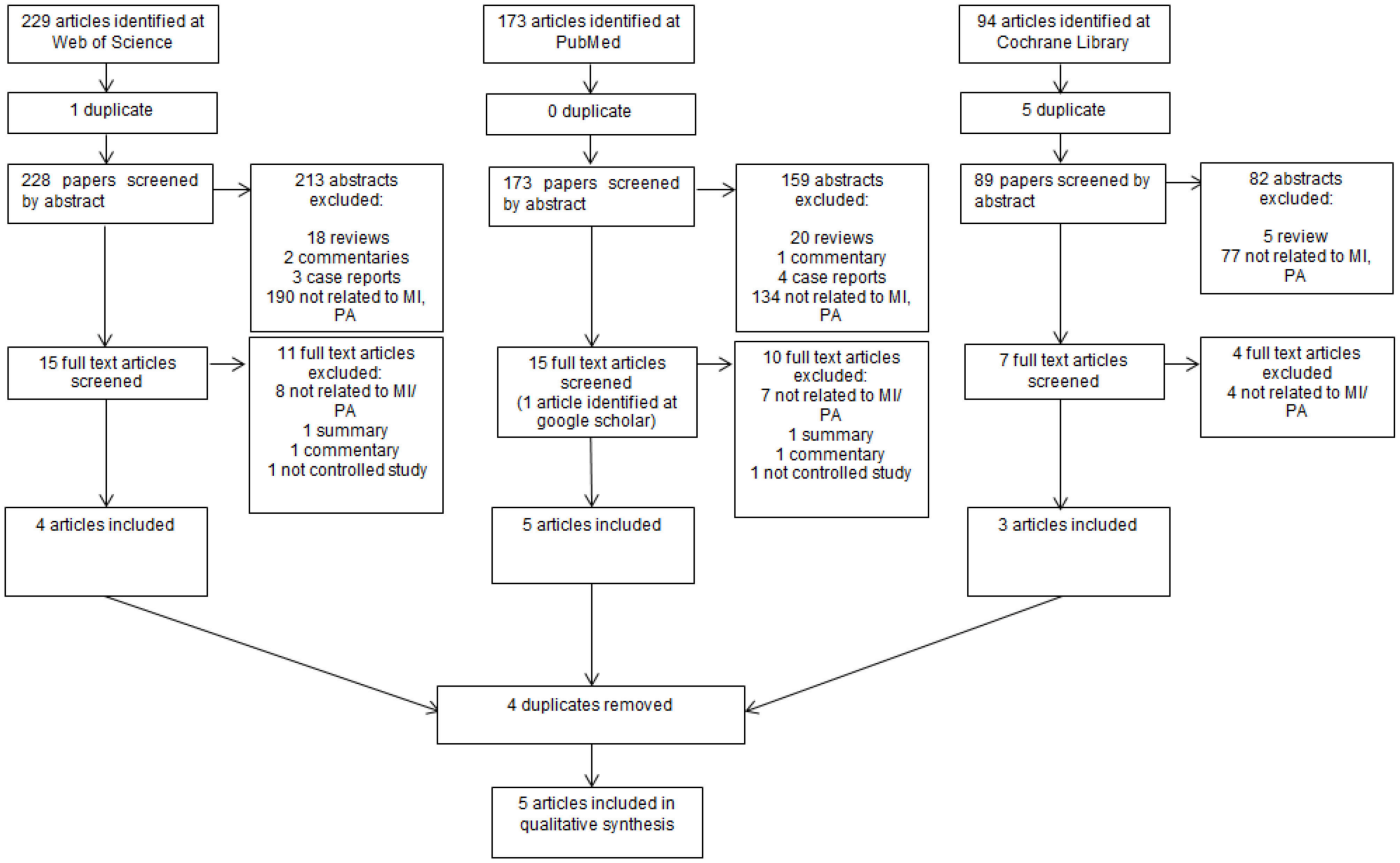
**Flow chart of the literature screening**.

In total the review yielded 496 articles. Forty Three Reviews, five commentaries, two summaries, two not controlled studies, and seven case reports were excluded. After screening titles and abstracts a further 454 articles were excluded. These papers did not involve both inflammatory and plaque indices in combination with motivational interviewing. Regarding the exclusion criteria, in total five papers could be included (Figure 1).

### Risk of bias

The risk of bias assessment ranged from 72 to 88% (Table 2). As in the risk of bias is shown, all studies did well in the evaluation. Main limitations were the insufficient presentation of inclusion- and exclusion-criteria and the number of counselors. A complete list of all included and excluded articles is presented in the supplementary material (Data Sheet 1).

### Study characteristics and outcomes

Three out of the five studies showed that the clinical periodontal values changed significantly in favor of the experimental group using MI as an adjunct to periodontal therapy (Table 3). One study showed a lower BOP in the experimental group compared to the non-MI control group (Jönsson et al., 2010). In two studies gingival inflammation decreased significantly higher in the experimental group than in the control group (Jönsson et al., 2009b; Woelber et al., 2016). In two studies, plaque values showed a significantly higher reduction in the experimental group compared to the control group (Jönsson et al., 2009b, 2010). The combination of multiple MI sessions and long-term study follow up of the patients achieved the best results in a non-surgical periodontal therapy referring to BOP and PI (Jönsson et al., 2009b, 2010). The study by Woelber et al. (2016) showed a positive effect of MI on plaque values and gingival inflammation with several MI sessions and a treatment period of 6 months. However, the studies with single MI interventions (Stenman et al., 2012; Brand et al., 2013) showed no significant differences in periodontal therapy.

**Table 3:** **Characteristics of included studies**.

**Author Year of publication**	**Woelber et al. (2016)**	**Brand et al. (2013)**	**Stenman et al. (2012)**	**Jönsson et al. (2010)**	**Jönsson et al. (2009b)**
n of patients in total	155	56	44	113	113
n control group	99	27	22	56	56
n experimental group	73	29	22	57	57
Background	Workshop in MI for dental students additional to non-surgical periodontal therapy	One MI-intervention additional to periodontal therapy	One MI-intervention additional to periodontal therapy	ITOHEP additional to periodontal therapy	ITOHEP additional to periodontal therapy
Experimental group	Periodontal therapy treated by students trained in MI	One MI-intervention before periodontal therapy	One MI-intervention before periodontal therapy	ITOHEP + periodontal therapy	ITOHEP + periodontal therapy
Control group	Periodontal therapy treated by students without training in MI	Periodontal therapy without additional MI intervention	Periodontal therapy without additional MI intervention	Periodontal therapy without additional MI intervention	Periodontal therapy without additional MI intervention
Type of MI	n.a.	Miller and Rollnick (2002)	Miller and Rollnick (1991, 2002)	Combination of MI Miller and Rollnick (2002) and cognitive behavioral principles (Bandura, 1977; Baranowski et al., 2002)	Combination of MI Miller and Rollnick (2002) and cognitive behavioral principles (Bandura, 1977; Baranowski et al., 2002)
Number and profession of counselors	56 dental students	1 MI trained counselor	1 psychologist	2 dental hygienists	2 dental hygienists
MI dose	4–5, re-evaluation after 4–6 weeks (duration 2–3 h); SPT: 1 appointment	Once after one week (15–20 min)	One MI-intervention before periodontal therapy (44 min)	Once a week at initial dental treatment, after SRP every third month (3,6,9,12 month)	Once a week at initial dental treatment, after SRP every third month (3,6,9,12 month)
Clinical measurements	PI, GI, PPD, CAL, BOP	BOP, PI, PPD	BOP, PI	PPD, BOP, PI	GI, PI
Number and profession of clinicians	56 dental students	2 dental hygienists	4 dental hygienists	1 periodontal specialist: clinical measurements 1 dental hygienist: treatment of periodontitis	n.a.
Follow-up	0–6 weeks	6, 12 weeks	2, 4, 16, 26 weeks	3 and 12 months	3 and 12 months
Type and duration of periodontal therapy	Systematic non-surgical periodontal therapy; SPT, ca 4–5 appointments of 2–3 h	Supportive periodontal therapy; n.a. duration	Systematic non-surgical periodontal therapy, 4 appointments of 1 h	Systematic non-surgical periodontal therapy, 4–5 appointments; n.a. duration	Systematic non-surgical periodontal therapy, 4–5 appointments of SRP; n.a. duration
Drop-Outs	21	3	5	n.a.	6
Outcome measurements	GI, PPD ↓ SWE IDR ↑MITI ↑	No difference between trialgroups	No difference between trialgroups	BOP, PI ↓	GI, PI ↓
Main findings	Teaching students in MI showed a significant effect on ID-cleaning self-efficacy in patients. MI-adherent communication was significantly improved in students.	The study showed no effects on oral hygiene and clinical outcomes with an additional brief MI intervention	The study showed no effects on oral hygiene and clinical outcomes with an additional brief MI intervention	ITOHEP was efficacious for improving gingival and periodontal inflammation in periodontal therapy.	ITOHEP was an efficacious in long term reduction of gingival and periodontal inflammation.

*Characteristics of included Studies. SPT, supportive periodontal therapy; GI, gingival values; PI, plaque values; BOP, bleeding on probing; PPD, probing pocket depth; ITOHEP, individually tailored oral health educational programme, MITI, motivational interviewing integrity code, SWE IDR, interdental cleaning self-efficacy; SRP, scaling and root planning*.

Except for one study (Brand et al., 2013), every study evaluated a systematic non-surgical periodontal therapy with additional MI. Woelber et al. (2016) reported about four to five sessions of periodontal therapy. Jönsson et al. (2009b, 2010) performed in their study four to five sessions of periodontal therapy. Stenman et al. (2012) treated the patients with four sessions, which lasted about 1 h. All treatments included initial phase with oral hygiene training, professional tooth cleaning, and SRP. The study by Brand et al. (2013) treated periodontal patients in the supportive periodontal therapy with unknown duration. Woelber et al. (2016) examined patients both in systematic periodontal therapy and supportive periodontal therapy. However, no further analysis between the treatment types was given. The studies showed different periods of study follow-up: Two studies (Brand et al., 2013; Woelber et al., 2016) examined patients 4–6 weeks after completion of the periodontal therapy. Stenman et al. (2012) reevaluated the patients after 2, 4, 16, and 26 weeks. Jönsson et al. (2009b, 2010) examined the clinical assessments after 3, 6, and 12 months. Regarding the MI interventions, in two studies patients received a single session of MI before the systematic periodontal therapy or during SPT (Stenman et al., 2012; Brand et al., 2013). In three studies the patients received multiple MI sessions (Jönsson et al., 2009b, 2010; Woelber et al., 2016).

Regarding the type of counselor, one study included MI sessions conducted by a psychologist specifically trained in MI (Stenman et al., 2012). In three studies the interventions were carried out by in MI trained dental hygienists (Jönsson et al., 2009b, 2010) or in MI trained dental students (Woelber et al., 2016). One further study engaged a consultant trained in MI (Brand et al., 2013). The definition of “consultants” and the extent to which they were trained in MI were not described in detail.

## Discussion

### MI as an adjunct to periodontal therapy

According to the analysis of the studies by Woelber et al. (2016) and Jönsson et al. (2009b, 2010), MI seems to have a positive influence on oral hygiene, periodontal inflammation and psychological oral hygiene factors in periodontal therapy. However, the available data is rather weak. Three out of five studies showed a positive outcome regarding the effects of additional MI interventions in periodontal therapy, while two studies showed no significant effects (Stenman et al., 2012; Brand et al., 2013). Amongst the three studies with positive outcomes, two studies embedded MI in a combination with other behavioral principles like the theory of self-efficacy (Jönsson et al., 2009b, 2010) and one study only found effects on psychological oral parameters (Woelber et al., 2016). Due to this low body of evidence there remains a need for further well-conducted long-term studies.

Nonetheless, no negative results for MI could be found and the shown effects were important factors, because reduced levels of plaque and gingival inflammation determine the long-term success of periodontal therapy (Axelsson et al., 2004; Eickholz et al., 2008). Furthermore, the application of MI seems to not be more time consuming than traditional techniques (Woelber et al., 2016).

### Duration of the follow-up

Regarding the question how the duration of study follow-up measures in MI-studies is important for the long-term effect, Jönsson et al. (2009b, 2010) conducted a study over a longer term within 12 months, compared to the other studies. These studies showed a significant reduction of BOP, GI, and PI in the MI-group. This positive effect of long-term studies regarding MI might be explainable due to several factors in clinical treatment of periodontal disease, because clinical interventions have a strong short-term effect (like scaling and root planing and professional mechanical plaque removal). Scaling and root planing (SRP) is very effective in the reduction of elevated pocket depths in addition to positive effects on the composition of the subgingival biofilm (Ramfjord et al., 1982; Axelsson et al., 2004; Goodson et al., 2012). Furthermore, sufficient oral hygiene is an important factor in reducing pocket depth values additionally to scaling and root planing (Nyman et al., 1977; Westfelt et al., 1998). A review by Needleman et al. (2015) found out that repeated oral hygiene instructions can achieve comparable results as repeated professional mechanical plaque removal alone. Thus, it is to be expected that behavioral interventions need a longer follow-up in periodontal studies to show their potential effects. This assumption is supported by Eickholz et al. (2008), who found that the most important risk factor for tooth loss after 10 years was the irregular participation of patients to supportive periodontal therapy. Therefore, it would be important to carry out long-term studies in order to investigate the effect on the motivation of patients to perform oral hygiene and to participate in supportive periodontal treatment regularly. Possible long-term studies could be performed by groups of trained and non-MI-trained dental professionals in separated dental practices. This would also give an impression if MI would be a feasible method in the dental practice and not only in the university setting.

### Kind of used MI in interventions

Data analysis revealed a lack of precise information about the kind of MI-interventions in the included studies. Even though there were only minor changes, the definition of Motivational Interviewing changed over the years (Miller and Rollnick, 1991, 2002, 2012). Additionally, due to the variety of methods and principles in MI (e.g., development of discrepancy, client-centered principles, evocation), the style of MI can differ amongst the counselors. For this, studies with a high number of counselors and a communication manual would be advisable to create an identical framework and to heighten the quality of the study. This manual should also relate to oral hygiene measurements and procedures (like demonstration by the professional and patient himself). Furthermore, the application of MI should not be combined with other behavioral interventions like in the studies by Jönsson et al. (2009b, 2010), even though some principles might be also included in MI framework (e.g., the theory of self-efficacy).

### Evaluated outcomes

In order to receive a comprehensive impression of the periodontal outcomes, only studies were included that investigated at least one inflammatory index and one plaque index (Mombelli et al., 2014). The plaque index serves as an indicator of plaque accumulation and the inflammatory index serves as a long-term parameter for the inflammatory status of the periodontium (Löe et al., 1965). In the recent review of Gao et al. (2014), partly relating to MI and the influence on oral hygiene and improving periodontal health, some of the mentioned studies had to be excluded in this review due to the lack of plaque or gingival indices. Within these studies, one study examined only the knowledge regarding oral hygiene and the self-efficacy in the patients (Stewart et al., 1996). Lalic et al. (2012) evaluated only the BOP of patients. Two studies investigated only plaque values (Almomani et al., 2009; Godard et al., 2011). Thus, the review by Gao et al. (2014) concluded studies with indices which allow only a limited view about the effect of MI on periodontal therapy with no risk of bias analyzation regarding the periodontal therapy. In this current review, the studies of Godard et al. (2011) and Almomani et al. (2009) were primarily included in the literature analysis and finally excluded due to the exclusion criteria (Table 1). Another interesting aspect of the use of MI as an adjunct to periodontal therapy is the broader influence on periodontal risk factors besides oral hygiene. In this context, a study by Schoonheim-Klein et al. (2013) showed a higher effort in smoking cessation of dental students trained in MI compared to non-trained students.

### Evaluation of MI-quality

From the five included studies, only two studies evaluated and controlled the application and quality of the MI elements (Stenman et al., 2012; Woelber et al., 2016). In the study of Woelber et al. (2016) the MI-trained students attained in average 3.35 points according to German version of the MITI 2.0 (Moyers et al., 2005; Brueck et al., 2009). The students of the control group achieved an average of 1.76 points in this assessment. According to Moyers et al. (2005), the beginning proficiency for MI begins with an average of five points and competency with six points and more. Stenman et al. (2012) performed the MI interventions by a trained psychologist in order to achieve a high quality of used MI elements. According to the MITI 3.0 coding system, average values of 2.5–3.5 points were achieved. The applied MITI 3.0 according to Moyers et al. (2007) defines competency in MI with values above four points. It should be mentioned that the rating scales of MITI 3.0 and MITI 2.0 are not comparable. However, the fidelity measurement allows an impression about the applied quality of MI and Miller and Rollnick (2014) stated that these information are essential when studies regarding MI are presented. Looking at the MITI values of Woelber et al. (2016) and Stenman et al. (2012) it seems like that in both studies MI was not applied in its full potential. Furthermore, Stenman et al. (2012) did present the values in general and not in detail for each MITI element in their publication.

### Counselor background

In addition, it has to be discussed how the professional background of the MI counselor influences the periodontal outcomes. Here, a single MI intervention by a psychologist showed no effect on clinical results in periodontal therapy (Stenman et al., 2012) and in studies with positive MI-outcomes MI was applied by MI-trained dental caregivers (Jönsson et al., 2009b, 2010; Woelber et al., 2016). It can be speculated that patients show more commitment to a health-specific counselor. This might be due the important role of empathy between the patient and the clinical examiner (Walseth and Schei, 2011). A good patient-examiner relationship automatically creates a certain motivation and is important for a long-term adherence of the patient. These assumptions also reveal an inherent bias when investigating the effects of MI in a certain health setting: the strength of the Stenman et al. study regarding the “clean” application of MI by an external psychologist also might weaken the possible effects in a “real” dental setting with a combined MI- and dental-expert.

Looking more closely at the study plans, all studies were conducted in a parallel-group design. Only the study by Woelber et al. (2016) treated the patients of the experimental- and control-group in a staggered interval, with the idea, that the therapists and patients could not interchange with each other. It should be noted that the seasonal treatment could have a positive or negative impact on the patients and the treatment of the students.

## Limitations

A general limitation is that no study conducted a “sham exposure.” The experimental groups received one or more MI sessions during periodontal therapy, and the control groups received periodontal therapy without additional MI interventions. It should be noted that a control group with training in non-MI-conversation (sham exposure) would be useful in order to avoid a Hawthorne effect of the trainees and the patients (Roethlisberger and Dickson, 1939).

Although the review beyond PubMed is not necessarily beneficial (Halladay et al., 2015), using more than three databases could possibly provide more studies.

## Summary

Taking all of the above under consideration, some important recommendations can be made for future studies:

- Due to the strong short-term clinical effects of scaling and root planing, antibiotics, and professional tooth cleaning, long-term studies are needed to evaluate the effect of MI on periodontal-related health behavior (like oral hygiene, smoking cessation, nutritional change, or patient adherence).
- A standard communication manual should be established in order to control and evaluate the used MI elements and oral hygiene instructions.
- A high number of dental professionals as study counselors should be trained in MI to a proficiency level to ensure effective use of MI and to avoid personal influences (like sympathy, friendliness etc.).
- Although the studies showed no deterioration of the clinical results due to the efficacy of SRP, several MI interventions seem to have a greater effect on the behavioral change of the patients (Martins and McNeil, 2009).

## Conclusions

This systematic review showed that the use of Motivational Interviewing as an adjunct to periodontal therapy might have a positive influence on clinical periodontal parameters and psychological factors related to oral hygiene. Due to the low body of evidence further long-term studies are needed.

## Author contributions

SK contributed to planning and conduction of the study and writing the manuscript. CR contributed to planning and conduction of the study and writing the manuscript. PR contributed to conduction of the study and writing the manuscript. JW contributed to planning and conduction of the study and writing the manuscript.

## Funding

The study was based on institutional funding. The article processing charge was funded by the German Research Foundation (DFG) and the Albert Ludwigs University Freiburg in the funding programme Open Access Publishing.

### Conflict of interest statement

The authors declare that the research was conducted in the absence of any commercial or financial relationships that could be construed as a potential conflict of interest.
