# Supplementary material for: Motivational Interviewing As an Adjunct to Periodontal Therapy—A Systematic Review
Source: Front Psychol. 2017 Feb 28;8:279. doi: 10.3389/fpsyg.2017.00279 (PMC5329060; doi:10.3389/fpsyg.2017.00279)
Supplement: Supplementary file 2 [file DataSheet2.DOCX]

| Risk of Bias | 0 Score | + 1 Score | n.a. |
| --- | --- | --- | --- |
|  |  |  |  |
| Type of MI  Number of Counsellors  MI training of Counsellors | Combination of several behavioral principles  Low number  No | Used version of MI regarding Miller/ Rollnick  High number  Yes | not mentioned  not mentioned  not mentioned |
| Fidelity measure | No monitoring of interventions | Monitoring of interventions | not mentioned |
|  |  |  |  |
| MI Dose  Number of Interventions  Profession of Counsellors  Quality of MI  Recruitment of patients  Type of periodontal therapy  Outcome measures  Calibration  Check of medication  Report of oral hygiene  Report of periodontal risk factors  Study follow up  Randomization  Blinding  Inclusion-/ Exclusion criteria  Participants (exp.-/control group)  Definition of trial groups  Number and profession of clinicians  Drop Out  Funding  Results | Duration of MI in combination with periodontal treatment mentioned  One  No dental professional or psychologist  No evaluation of used MI (e.g. MITI)  Not comprehensible  Partly mentioned  Selective report  No  No  No  No  Short follow up (a few weeks)  No  No  Missing data  Different numbers  Not comprehensible  No dental professionals  No  No  Missing data | Duration of MI mentioned  More than one  Psychologist, dental professional  Evaluation of used MI (e.g. MITI)  Mentioned  Fully mentioned  All measurements reported  Yes  Yes  Yes  Yes  Long term follow up (ca. 12month)  Yes  Yes  comprehensible  Same quantity  comprehensible  Dental professionals  Yes  Yes  Complete data | not mentioned  not mentioned  not mentioned  not mentioned  not mentioned  not mentioned  not mentioned  not mentioned  not mentioned  not mentioned  not mentioned  not mentioned  not mentioned  not mentioned  not mentioned  not mentioned  not mentioned  not mentioned  not mentioned  not mentioned  not mentioned  not mentioned |
|  |  |  |  |
| Total Score:  Maximum Score:  Percentage: |  |  |  |
